# Visual hallucinations in neurosurgery: A systematic review and two case insights into Charles Bonnet Syndrome

**DOI:** 10.1016/j.bas.2025.104257

**Published:** 2025-04-24

**Authors:** Erica Grasso, Giulio Bonomo, Francesco Certo, Giacomo Cammarata, Giuseppe M.V. Barbagallo

**Affiliations:** a Department of Neurological Surgery, Policlinico "G. Rodolico-S. Marco" University Hospital, Catania, Italy; b Interdisciplinary Research Center on Brain Tumors Diagnosis and Treatment, University of Catania, 95123, Catania, Italy; c Department of Medical and Surgical Sciences and Advanced Technologies "G.F. Ingrassia", University of Catania, Catania, Italy

**Keywords:** Brain tumors, Charles Bonnet syndrome, Neurosurgery, Postoperative complications, Visual hallucinations

## Abstract

**Introduction:**

Visual disturbances in brain tumor patients occur as a direct consequence of tumor location or as a complication after surgery. These can range from minor vision changes to severe impairments, including visual hallucinations.

**Research question:**

Charles Bonnet Syndrome (CBS) is an underrecognized cause of complex visual hallucinations in patients with visual system disorders, particularly following neurosurgical interventions. We present two cases of CBS following neurosurgical procedures, recruited from our department.

**Materials and methods:**

Detailed patient histories, neurological and ophthalmologic evaluations, magnetic resonance imaging (MRI), computerized perimetry, electroencephalogram (EEG), and psychiatric assessments were conducted. A systematic literature review was also performed, focusing on CBS in the context of neurosurgery.

**Results:**

The literature review included 13 studies with 17 patients diagnosed with CBS related to neurosurgery. The average age was 54.8 ± 16.5 years. CBS was associated with various pathologies, including mesial temporal lobe sclerosis, glial tumors, and pituitary adenomas. Most CBS cases (76 %) developed post-surgery. CBS onset was linked to extra-axial tumors causing direct mechanical compression of the optic chiasma or nerve, which often resolved completely after tumor excision. Intra-axial tumors were more likely to cause CBS post-operatively, likely due to surgical trauma affecting the optic tracts or visual cortex, and often required additional treatment.

**Discussion and conclusion:**

CBS is frequently underrecognized in neurosurgical patients. Differentiating between extra-axial and intra-axial tumor involvement is critical for appropriate treatment. Standardized diagnostic and treatment protocols are needed to improve outcomes, and further research is essential to better understand CBS mechanisms in neurosurgical contexts.

## Highlights

• Differentiating extra-axial and intra-axial tumors is key to CBS treatment.
• Extra-axial CBS often resolves post-excision; intra-axial requires further care.
• CBS is underrecognized, highlighting the need for standardized neurosurgical protocols.

## 1 Introduction

Visual disturbances are a common issue associated with brain tumors, both as a direct consequence of the tumor's location and as a complication following surgical intervention. These disturbances can range from subtle changes in vision, such as blurring or double vision, to more severe impairments, including complete loss of vision or complex visual phenomena like hallucinations. The involvement of critical areas of the brain responsible for processing visual information, such as the occipital lobe, optic pathways, or associated structures, can lead to these symptoms. Additionally, post-operative complications, such as swelling or damage to nearby brain tissue, can exacerbate these visual disturbances (Bowman et al., 2023). Differentiating the underlying causes of visual hallucinations is often challenging but essential for accurate diagnosis and appropriate treatment.

Among the different types of visual hallucinations, Charles Bonnet Syndrome (CBS) is particularly noteworthy, yet it is often underrecognized by neurosurgeons. CBS, often referred to as the "phantom image" syndrome, is characterized by recurrent or persistent complex visual hallucinations in individuals with visual system disorders who maintain intact insight, intellectual capacity, and normal cognitive functioning, without any underlying psychiatric conditions (Aslan et al., 2023; Carpenter et al., 2019).

Historically, CBS was considered rare, with only a few cases reported in the literature before 1990. However, recent studies indicate that 41 %–59 % of visually impaired patients experience visual phenomena, with 11–15 % exhibiting complex hallucinations due to a medical condition or artificial causes (Aslan et al., 2023). These prevalence figures are likely underestimated due to limited awareness of CBS among physicians and the absence of standardized diagnostic criteria. Although there is no universal consensus on diagnostic criteria, Hamedani et al. (2019) suggest that the presence of visual hallucinations combined with visual loss, normal cognitive function, and preserved insight are essential for diagnosing CBS (Hamedani and Pelak, 2019). Conditions such as epilepsy, Parkinson's disease, diffuse Lewy body disease, migraine, stroke, other central nervous system lesions, encephalopathy, substance abuse, and narcolepsy are specifically mentioned as exclusion criteria (Carpenter et al., 2019; Hamedani and Pelak, 2019).

Despite the growing body of research on CBS, its association with neurosurgical pathologies has not been systematically explored. In this context, we present two cases of CBS associated with neurosurgical conditions and provide a systematic review of the literature on neurosurgical pathologies linked to the onset of CBS.

## 2 Materials and methods

In the illustrative cases presented, we recruited patients from the Department of Neurosurgery at the Azienda Ospedaliero-Universitaria Policlinico "G. Rodolico-San Marco" in Catania, Italy. Detailed patient histories were gathered, along with comprehensive neurological and ophthalmologic evaluations. Diagnostic imaging, including pre- and post-operative magnetic resonance imaging (MRI), was utilized to assess brain lesions and surgical outcomes. Computerized perimetry confirmed visual field deficits, while electroencephalogram (EEG) excluded epileptiform activity. Psychiatric assessments evaluated the presence of psychiatric comorbidities and the need for treatment of visual hallucinations. The data were systematically collected and reported in accordance with the CARE guidelines, ensuring clarity and completeness in the analysis of these cases.

### 2.1 Literature review search strategy

**Fig. 1:**
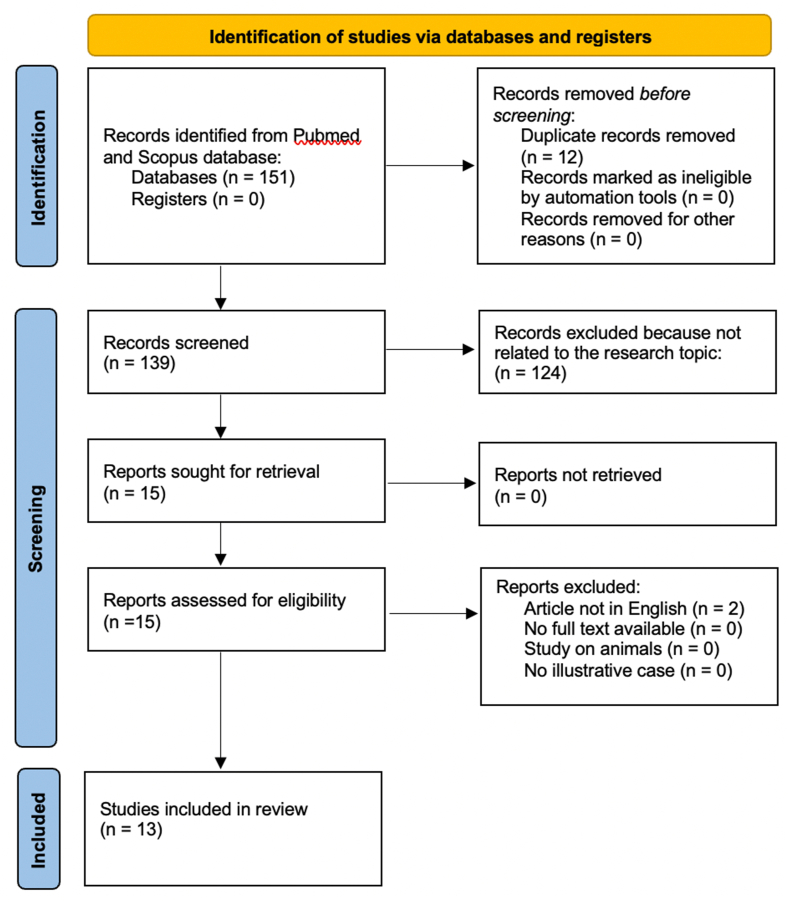
PRISMA 2020 flow diagram for new systematic reviews which included searches of databases and registers only.

An extensive search of the English medical literature was conducted using multiple electronic databases, including MEDLINE/PubMed and Scopus. The search strategy employed medical subject heading (MeSH) terms such as “Charles Bonnet Syndrome,” “CBS,” and “Neurosurgery,” in various combinations. References were selected based on the scope of this review and in accordance with PRISMA (Preferred Reporting Items for Systematic Reviews and Meta-Analyses) guidelines (Fig. 1).

Two authors (EG, GB) independently evaluated each record. The selection of search terms was informed by analyzing the titles, abstracts, and subject indexing of known relevant studies. After identifying candidate search terms, the researchers independently screened the titles and abstracts of all retrieved articles. In cases of disagreement, a consensus was reached through discussion, and if necessary, a third researcher (FC) was consulted to make the final decision. Subsequently, EG and GB independently reviewed the full-text articles for inclusion. Again, any disagreements were resolved by discussion, with FC providing a final decision if needed.

All publication types were eligible for inclusion, with no time restrictions applied to the inclusion/exclusion criteria. The initial search yielded 151 publications, of which only 13 were deemed relevant. These selected articles included case reports, reviews, and systematic reviews of the literature.

Data collection focused on the following details.

1. Study author and year
2. Patient age and sex
3. Positive history of pre-operative seizures
4. Pathology and surgical procedure (if applicable)
5. Associated visual deficits and their onset
6. Features of interictal and hallucinatory EEG
7. Characteristics and descriptions of hallucinations
8. Duration and outcomes after conservative treatment

The methodological quality of the included studies was assessed based on.

- Inclusion criteria: diagnosis of CBS, underlining neurosurgical pathology, visual deficit description
- Exclusion criteria: article not in English, no full text available, study on animals, no illustrative case

## 3 Illustrative cases

### 3.1 Case 1

A 62-year-old female was referred to our department for a recurrence of right temporal glioblastoma, detected during clinical-radiological follow-up. The patient had previously undergone surgical tumor excision with complete resection and received adjuvant therapy according to STUPP protocol, resulting in a progression-free survival (PFS) of 35 months (Stupp et al., 2005).

At admission, the patient presented with a left superior homonymous quadrantanopia, confirmed by pre-operative ophthalmologic evaluation through computerized perimetry. Her medical history includes hypothyroidism, osteoporosis, and fibromyalgia.

**Fig. 2:**
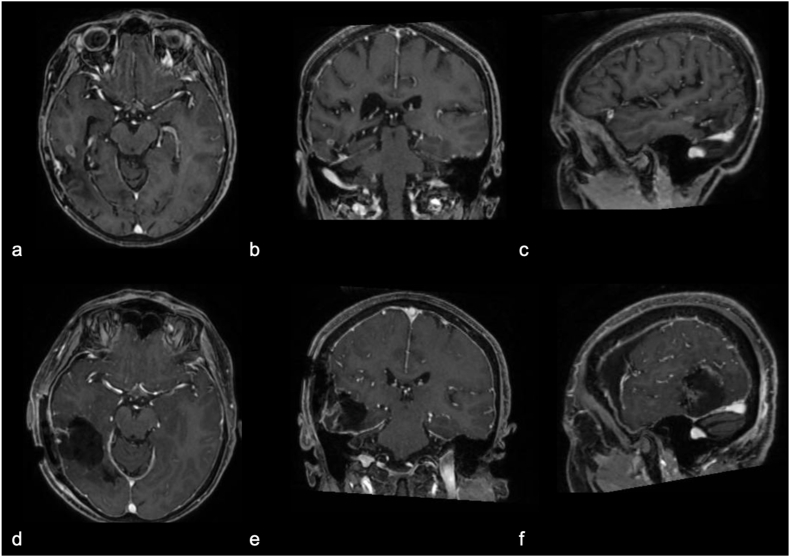
Preoperative brain MRI images, T1-weighted sequences with gadolinium, showing (a) Axial, (b) Coronal, and (c) Sagittal views of the recurrence of a right temporal glioblastoma; postoperative images (d, e, f) after complete tumor resection.

A second surgery was performed, and a post-operative Gadolinium-enhanced MRI showed gross total resection (Fig. 2). On the third postoperative day, the patient reported vivid visual hallucinations, such as insects, gears, and silhouettes of acquaintances, primarily in the left visual hemifield. Her insight remained intact. An EEG showed no epileptiform abnormalities. After psychiatric consultation, Risperidone 2 mg/day was prescribed. The patient was discharged on day 28 with reduced visual hallucinations, though they persisted.

### 3.2 Case 2

**Fig. 3:**
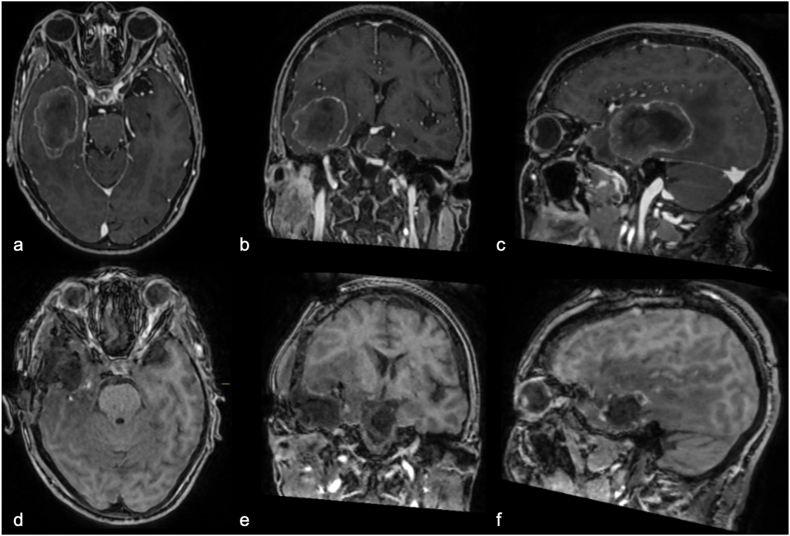
Preoperative brain MRI images, T1-weighted sequences with gadolinium, showing (a) Axial, (b) Coronal, and (c) Sagittal views of metastasis in the left temporal lobe; postoperative images (d, e, f) after complete tumor resection.

A 77-year-old female presented to our department with a 3-month history of temporal disorientation, mood changes, and visual agnosia. She had no prior psychiatric history but had previously undergone surgery and chemotherapy for colon carcinoma. Neurological examination revealed a left superior homonymous quadrantanopia, confirmed by pre-operative ophthalmologic evaluation with computerized perimetry. MRI suggested a possible metastasis in the left temporal lobe (Fig. 3a and b-c). The patient underwent surgical resection, with a post-operative MRI showing no residual tumor (Fig. 3d and e-f). The final histopathological diagnosis was cerebral metastasis from a low-grade (moderately differentiated, G2) intestinal-type adenocarcinoma.

On the third post-operative day, she began experiencing visual hallucinations, such as seeing soap bubbles and a child emerging from the floor in the upper left visual field. She remained aware that these hallucinations were not real and did not engage with them, maintaining intact insight throughout. An EEG showed no epileptiform abnormalities. The administration of Levetiracetam 500 mg twice daily did not alter the frequency or content of the hallucinations. A psychiatric consultation determined that no additional medical treatment was necessary, as the hallucinations were not distressing and did not significantly impact her quality of life. The patient was discharged on day 10, and the hallucinations spontaneously improved.

## 4 Results

The literature review included 13 studies with 17 patients (53 % female, 47 % male) diagnosed with CBS related to neurosurgical conditions (Table 1). The average age at diagnosis was 54.8 ± 16.5 years (range 29–84). CBS was attributed to mesial temporal lobe sclerosis in 11.7 % of cases, glial tumors in 11.7 %, pituitary adenomas in 23.5 %, supratentorial metastases in 17.6 %, post-trauma in 17.6 %, meningiomas in 11.7 %, and one case (5.8 %) of cavernous angioma. Epileptic seizures were observed in 29 % of cases, with EEGs performed in 18 % showing no consistent epileptiform patterns.

**Table 1:** Table summarizing the results of our systematic review on Charles Bonnet Syndrome in neurosurgery.

	Authors and year	Age and Sex	Pre-operative seizures	Pathology and Surgical Procedure	Neurological deficits and timing of onset	EEG	Hallucinations	Characteristics	Timing of onset	Duration and treatment
**1**	*Freiman et al., 2004*	40M	Partial seizures	**Ammon horn sclerosis -** amygdalohippocampectomy	Upper-left quadrantanopia (*post-operative)*	N/M	3D pyramids and spheres, which were spinning around their own axes	Hallucinations were restricted to the specific region of the visual field defect.	Post-op	4 days, complete remission following treatment with Topiramate
**2**	*Freiman et al., 2004*	29F	Complex partial seizures with secondary generalization.	**Ammon horn sclerosis -** amygdalohippocampectomy	Upper-left quadrantanopia (*post-operative)*	Normal	Intermittent moving human faces	Hallucinations were restricted to the specific region of the visual field defect.	Post-op	7 days, remission with Carbamazepine
**3**	*Freiman et al., 2004*	38F	Focal motor seizures	**Parietal astrocytoma** bordering the thalamus -	Lower-right quadrant-anopia (pre-operative), right hemianopia (*post-operative)*	Polymorphic slowing in the delta wave but no interictal spiking or ictal patterns	Multi-coloured human faces and moving bars	Intermittent visual hallucinations restricted to the specific region of the visual field defect.	Post-op	6 months, conservative treatment with Carbamazepine
**4**	*Freiman et al., 2004*	50F	Single grand mal seizure	**Left parietooccipital Glioblastoma**	upper-right quadrantanopia (pre-operative); right homonymous hemianopia (*post-operative)*	N/M	Flowering 3D plants	Intermittent visual hallucinations restricted to the specific region of the visual field defect.	Post-op	21 days, conservative treatment with Carbamazepine
**5**	*Contardi et al., 2007*	49F	Generalized tonic-clonic, autonomic	**Cavernous angioma in the mesial portion of the left temporal lobe** - left antero-mesial temporal lobectomy	Right hemianopia *(post-operative)*	Left temporal spikes and sporadic bursts of theta activity in the left anterior temporal leads	Coloured “Lilliputian” figures of women and children, either static or moving, but usually running in meadows or even lying in bed with her, or, occasionally, brightly coloured countryside scenes	Disappeared with eyes closing	Post-op	2 months, spontaneous complete regression
**6**	*Hashemi et al., 2012*	84F	No	**Pituitary adenoma** with suprasellar extension, compression of the optic chiasm and invasion of the left cavernous sinus – Transsphenoidal resection	Bilateral visual acuity impairment (*pre-operative)*	N/M	Children unknown to patient	N/M	Pre-op	Complete resolution after surgery
**7**	*Gander et al., 2013*	68M	No	Dislocated **left orbital floor fracture** - Surgical revision by a transconjunctival approach, titanium mesh insertion and Frost suture.	Visual acuity of 0.6 in the affected and 0.3 in the contralateral eye (*pre-operative)*	N/M	Shaped figures creeping across the room.	N/M	Post-op	Complete remission 2 h after removal of the Frost suture
**8**	*Paradowski et al., 2013*	65M	No	**Metastasis left occipital lobe**	Paracentral scotoma in the visual field related to the blind spot, located in the lower temporal quadrant, and lower sensitivity of the peripheral retina (*Pre-operative*)	N/M	Ghost image, people with “second lips,” and “flower-like” and “lamp-like” images.	N/M	Pre-op	N/M
**9**	*Park et al., 2016*	46M	No	**Pituitary macroadenoma** - Transsphenoidal resection	Limited lateral gaze of the right eye with diplopia. *(pre-operative)*	N/M	A black and white image of a giant sea anemone moving regularly and slowly. Colourful animated images such as a desert, sculptures, fishes, curtains, and flower-like forms.	Disappeared with eyes opening	Post-op	2 weeks
**10**	*Kosman et al., 2017*	52F	No	**Olfactory groove meningioma** – surgical excision	Bilateral optic nerve atrophy, complete visual loss in left eye *(pre-operative)*	N/M	Men with black snakes coming out of their bodies.	No specific spatial distribution	Pre-op	Complete resolution after surgical mass removal
**11**	*Diana et al., 2021*	43M	No	**Frontal Meningioma –** partial resection	Bilateral amaurosis *(pre-operative)*	N/M	Ex-partner spoke to him and constantly warned him about his health, visiting him frequently in the hospitalization area	No specific spatial distribution	Pre-op	No resolution
**12**	*Irizarry et al., 2022*	42M	No	**Previous Traumatic brain injury** on blind patient affected by retinitis pigmentosa	Bilateral eye blindness secondary to retinitis pigmentosa *(pre-operative)*	N/M	Appearance of children standing around his household, followed by the later appearance of children running around the same household	Intermittent. No specific spatial distribution	/	4-day, complete resolution after conservative treatment with Olanzapine
**13**	*Ghabi et al., 2023*	44F	No	**Pituitary macroadenoma** - Transsphenoidal resection	Temporal quadrantanopia *(pre-operative)*	N/M	Unfamiliar human faces	N/M	Pre-op	4 months complete resolution after conservative treatment (Olanzapine)
**14**	*Aslan et al.,* *2023*	81F	No	**Optic atrophy due to recurrent pituitary macroadenoma** – Conservative treatment	Visual acuity impairment (*pre-operative)*	N/M	Simple geometric shapes to well-formed complex “human-like figures.” Within a year, the visual experiences became more apparent, with the human-like figure suddenly appearing, walking alongside in female clothes and shapes before they disappeared abruptly. Insight was preserved	N/M	/	Partial resolution with Haloperidol
**15**	*Ovchinnikov et al., 2023*	74M	No	**Occipital lobe metastasis** – Surgical resection + post-operative radiotherapy	Right-sided hemianopia *(pre-operative)*	EEG did not reveal any epileptogenic activity	Patient recurrently viewed visual images from his past, such as seeing his father, grandfather, or his dog standing in the room, in the right field of vision only. He also reported additional perseveration of a previously seen image (palinopsia) and deformation of central vision (metamorphopsia).	Hallucinations occurred only in the right field of vision	Post-op	Complete resolution 18 months after surgery without any medications.
**16**	*Ovchinnikov et al., 2023*	76F	No	**Left parietal metastasis –** Surgical resection	Global aphasia and confusion, visual deficits not mentioned *(pre-operative)*	N/M	Patient saw a work colleague next to her, with whom she had worked decades previously. She also saw a pregnant woman sitting next to her bed. Once a day, she observed her late husband walk around her room	Hallucinations occurred in all visual fields.	Post-op	Death in the following weeks
**17**	*Wong et al., 2024*	51M	No	**Terson Syndrome resulting from frontoparietal traumatic subarachnoid hemorrhage (tSAH) with subdural hematoma (SDH) along the right parietal convexity -** Conservative	Bilateral non-light perceiving blindness concerning Terson Syndrome *(pre-operative)*	N/M	Patient visualized "three fighter jets that were laughing at him"; the patient works with fighter jets which give him insight into the hallucinations. He also stated that he could visualize holes and staircases while trying to walk, which made him feel unsteady. Additionally, when he was pushed into his wheelchair, he experienced a sensation of falling.	Hallucinations occurred in all visual fields.	/	Partial resolution with Valproic Acid
**18**	*Grasso et al., 2024 (****current study****)*	62F	No	**Right temporal glioblastoma recurrency –** Surgical resection	Left superior homonymous quadrantanopia with complete involvement of the central area *(pre-operative)*	N/M	Patient visualized insects, gears, and silhouettes of acquaintances interacting with her. Insight was preserved.	Hallucinations were predominantly restricted to the specific region of the visual field defect.	Post-op	Partial resolution with Risperidone.
**19**	*Grasso et al., 2024 (****current study****)*	74F	No	**Right temporal lobe metastasis –** Surgical resection	Upper left homonymous quadrantanopia	N/M	Patient visualized saw soap bubbles and a child coming out of the floor. Insight was preserved.	The hallucinations were predominantly restricted to the specific region of the visual field defect.	Post-op	Partial regression without any medications

EEG: electroencephalogram.

Hallucination content was diverse and not correlated with the disease's location or surgical treatment. In 41 % of cases, hallucinations were restricted to the visual field deficit, while in 18 %, they had no specific spatial distribution. In 12 % of cases, hallucinations disappeared when the eyes were open, and 29 % of reports did not specify any visual field involvement.

Most hallucinations (76 %) developed post-surgery, while 18 % were present at initial presentation, and 12 % of patients did not undergo surgery. Complete resolution of hallucinations occurred within a median of 17.5 days (IQR 8.75–100) with treatments, including Topiramate (6 %), Carbamazepine (18 %), and Olanzapine (12 %). Spontaneous regression was observed in 18 % of cases, and one case resolved rapidly after Frost suture removal. In 41 % of cases, only partial resolution was reported, with no specific duration of hallucinations provided.

Pre-operative CBS onset was predominantly associated with extra-axial tumors, while post-operative CBS onset was more commonly linked to intra-axial lesions.

## 5 Discussion

CBS is a condition characterized by complex visual hallucinations in individuals with significant vision loss (Diana et al., 2021). Despite the absence of standardized diagnostic criteria, CBS is typically identified by four key features: persistent complex visual hallucinations, insight into their unreality, absence of other types of hallucinations, and no delusions (Ghabi et al., 2023; Ffytche, 2005).

Three main theories attempt to explain the pathogenesis of CBS. The "sensory deprivation" or "deafferentation theory" suggests that loss of visual input leads to spontaneous brain excitation. The "release theory" associates CBS with excessive excitation and the consequent release of visual hallucinations, while the "irritative theory" involves distal injuries transmitting abnormal input to the visual cortex, leading to excitatory activity in the temporal and occipital lobes (Aslan et al., 2023; Ffytche, 2005; Contardi et al., 2007; Rojas and Gurnani, 2024).

Although the exact mechanisms of CBS remain debatable, the ventral visual pathway seems to play a key role. Sasaki et al. reported significant hyperactivation of the ventral visual pathway, particularly in the lateral occipitotemporal cortex, through 18F-fluorodeoxyglucose (FDG)-positron emission tomography (PET) (Sasaki et al., 2021). Allen et al. found a temporal correlation between visual hallucinations and activity in the ventral "object recognition" stream (Allen et al., 2008). Similarly, Adachi and colleagues reported hyperperfusion in the thalamus, striatum, and lateral temporal cortex during hallucinations, indicating involvement of the ventral visual stream (Carpenter et al., 2019; Adachi et al., 2000).

CBS prevalence remains unclear, with estimates ranging from 0.8 % to 15 % among patients with visual pathway alterations (Diana et al., 2021). Conditions like glaucoma, cataracts, and age-related macular degeneration are commonly associated with CBS, although any significant visual impairment can lead to the syndrome (Diana et al., 2021; Chen, 2014). Medications described in literature included antiepileptic and antipsychotics with variable benefits (Ghabi et al., 2023; Freiman et al., 2004). A systematic review by Hamedani and Pelak emphasized the diagnostic challenges of CBS due to its heterogeneous presentation, variability in hallucination characteristics, and vision loss severity (Hamedani and Pelak, 2019). This underscores the need for more standardized diagnostic criteria to improve clinical accuracy.

Our literature review, encompassing 13 studies and 17 patients, provided further insights into CBS related to neurosurgical conditions. While hallucination content was diverse, it did not correlate with the specific location of the disease or the type of surgical intervention.

In our experience, we documented two cases of CBS following neurosurgical interventions. One patient with recurrent right temporal glioblastoma experienced vivid visual hallucinations post-surgery, which were partially reduced with Risperidone. Another patient with a left temporal lobe metastasis reported hallucinations that spontaneously improved without significantly affecting her quality of life. It is crucial to note that visual disturbances associated with neurosurgical pathologies, including visual hallucinations, are often underdiagnosed and poorly managed. CBS is frequently misunderstood and incorrectly classified, complicating its study and the ability to draw firm conclusions about its mechanisms and treatment. This lack of recognition also hinders proper patient care and limits the ability to conduct comprehensive studies on CBS. In suspected CBS cases, clinicians should exclude psychiatric disorders, neurological diseases, metabolic abnormalities, and substance use as causes of visual hallucinations. Lack of insight might suggest alternative diagnoses, such as bipolar disorder, Alzheimer's disease, or Parkinson's disease (Chen, 2014). Thus, comprehensive patient history and a deep understanding of CBS are essential for accurate differential diagnosis.

Interestingly, our findings revealed that pre-operative CBS onset was predominantly associated with extra-axial tumors causing direct mechanical compression on the optic chiasma or optic nerve. In contrast, post-operative CBS onset was more commonly linked to intra-axial lesions, likely due to surgical trauma affecting the optic tracts, radiations, or visual cortex.

We hypothesize that CBS onset differences may relate to the type of compression exerted by lesions. In the extra-axial group, direct mechanical compression on the optic chiasma or optic nerve was observed, while intra-axial tumors typically caused white matter fiber displacement. CBS in these cases may not result from the lesion's location itself but as a secondary effect of surgical trauma that potentially injures the optic tracts, radiations, or visual cortex (Masjoodi et al., 2018).

This observation has crucial implications. We found that in cases where CBS was caused by direct mechanical compression (i.e., extra-axial tumor) near the optic chiasma, radical tumor excision often led to complete symptom remission (Ghabi et al., 2023; Hashemi et al., 2012; Gander et al., 2014; Park et al., 2017; Kosman and Silbersweig, 2018). A notable example is the case reported by Gander et al., where hallucinations acutely occurred after surgical revision of a dislocated left orbital floor fracture, and removal of the Frost suture led to hallucination regression just 2 h post-operation (Gander et al., 2014). Conversely, when CBS arose post-operatively, partial or complete remission of hallucinations often required administration of antiepileptic and antipsychotic medications (Contardi et al., 2007; Freiman et al., 2004; Ovchinnikov et al., 2024).

Our study is limited by the small sample size and the heterogeneity of the cases reviewed, which prevents definitive conclusions about the efficacy of specific treatments for CBS. Additionally, the retrospective nature of the literature review limits the ability to control for confounding variables. Future research should focus on larger, prospective studies to better understand CBS mechanisms, particularly in neurosurgical contexts. There is also a pressing need for standardized diagnostic criteria and treatment protocols to improve clinical outcomes and ensure consistent care across different settings.

## 6 Conclusions

CBS is often underrecognized in neurosurgical patients with visual impairment. Our findings indicate that CBS can occur both pre- and post-operatively, with a distinction between extra-axial and intra-axial tumor involvement. CBS associated with extra-axial tumors, which cause direct mechanical compression of the optic chiasma or nerve, often resolves completely after tumor excision. In contrast, CBS linked to intra-axial lesions is more likely due to surgical trauma affecting the optic tracts or visual cortex, requiring additional treatment for symptom management.

This distinction underscores the need for targeted interventions based on the type of tumor involvement and emphasizes the importance of standardized diagnostic and treatment protocols to improve patient outcomes. Further research is necessary to enhance our understanding of CBS, particularly in the context of neurosurgical conditions.

## Consent to participate

Informed consent was obtained from all individual participants included in the study.

## Consent to publish

The authors affirm that human research participants provided informed consent for publication of the images and data.

## Author contributions

All authors contributed to the study conception and design. Material preparation, data collection and analysis were performed by Erica Grasso and Giulio Bonomo. The first draft of the manuscript was written by Erica Grasso and Giulio Bonomo and all authors commented on previous versions of the manuscript. All authors read and approved the final manuscript.

## Ethics approval

This is an observational study. The University of Catania Research Ethics Committee has confirmed that no ethical approval is required.

## Funding

This research was partially funded by the Department of Medical and Surgical Sciences and Advanced Technologies "G.F. Ingrassia", University of Catania, Catania, Italy.

## Declaration of competing interest

The authors have no relevant financial or non-financial interests to disclose.
